# Supplementary material for: Preclinical Evaluation of Podoplanin-Targeted Alpha-Radioimmunotherapy with the Novel Antibody NZ-16 for Malignant Mesothelioma
Source: Cells. 2021 Sep 22;10(10):2503. doi: 10.3390/cells10102503 (PMC8533940; doi:10.3390/cells10102503)
Supplement: Supplementary file 1 [file cells-10-02503-s001.zip › cells-1351149-supplementary.pdf]

Supplementary Materials

Preclinical evaluation of podoplanin-targeted alpha-radioimmunotherapy with the novel antibody NZ-16 for malignant mesothelioma

Hitomi Sudo[1], Atsushi B. Tsuji[1], Aya Sugyo[1], Mika K. Kaneko[2], Yukinari Kato[2,3], Kotaro Nagatsu[4], Hisashi Suzuki[4], and Tatsuya Higashi[1]

Supplementary Methods

Immunofluorescence staining

H226 cells were seeded on coverslips, incubated overnight, and then fixed with 4% paraformaldehyde. Immunofluorescence staining was conducted using anti-PDPN antibody NZ-12 and NZ-16 as a primary antibody and Alexa Fluor 488 goat anti-mouse IgG (Thermo Fisher Scientific) as a secondary antibody. The coverslips were mounted in mounting medium with DAPI (Vector Laboratories, Burlingame, CA, USA). Fluorescence images were captured with an exposure time of 500 msec for PDPN and 25msec for DAPI using a fluorescence microscope BX53 (Olympus, Tokyo, Japan) and cellSens Standard software (ver. 1.7.1, Olympus).

Cell binding assay

H226 cells (1.3×10^6) in phosphate-buffered saline with 1% BSA were incubated with ^{225}Ac-labeled NZ-16 antibody on ice for 60 min. After washing, cell-bound radioactivity was measured using a gamma-counter using an energy window of 200–300 keV.

Histologic analysis

The spleen, kidney, liver and femur (bone marrow) were resected from mice on day 7 after injection with intact NZ-16 (0 MBq, n = 3/time-point), ^{90}Y-labeled NZ-16 (3.7 MBq, n = 3/time-point) and ^{225}Ac-labeled NZ-16 (18.5 kBq, n = 3/time-point). The organs were fixed in 10% neutral-buffered formalin and embedded in paraffin. The organs sections (1-µm thick) were deparaffinized and stained with hematoxylin and eosin (H&E).

TUNEL staining

H226 tumors were resected from mice on days 1, 3, and 7 after injection with intact NZ-16 (0 MBq, n = 3/time-point), ^{90}Y-labeled NZ-16 (3.7 MBq, n = 3/time-point) and ^{225}Ac-labeled NZ-16 (18.5 kBq, n = 3/time-point). The tumors were fixed in 10% neutral-buffered formalin and embedded in paraffin. Apoptosis was detected using the DeadEnd Colorimetric TUNEL System (Promega, Madison, WI, USA).

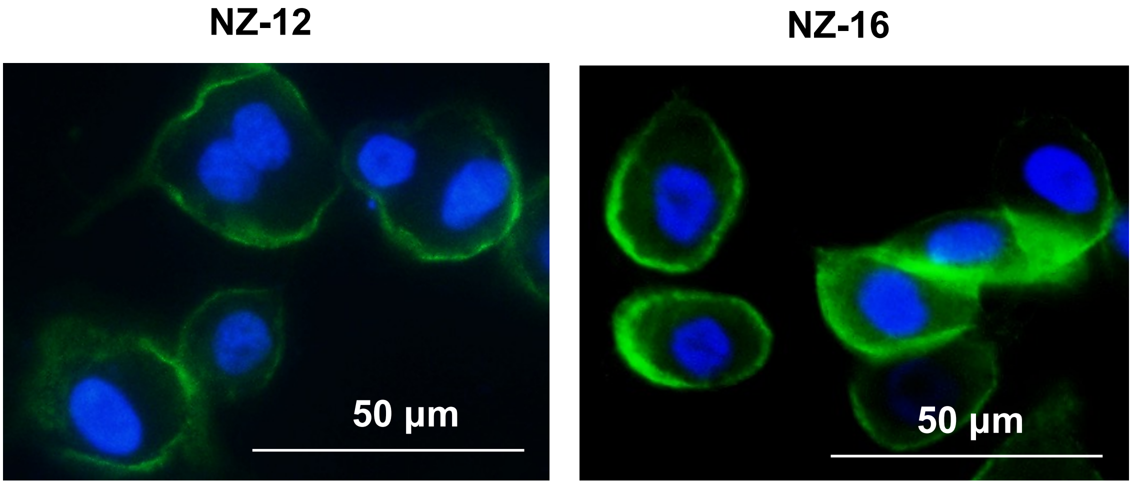

Supplementary Figure S1. Immunofluorescence staining of H226 cells using anti-PDPN antibody NZ-12 and NZ-16 (Green). The nuclei were counterstained with DAPI (blue).

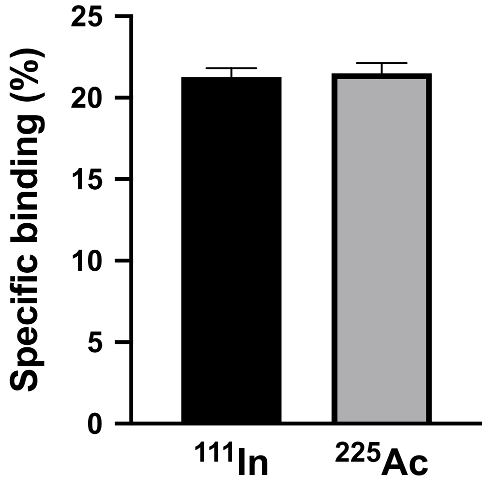

Supplementary Figure S2. Cell binding assay of ^{111}In- and ^{225}Ac- labeled NZ-16 with H226 cells (1.3 × 10^6 cells). Data indicate the mean and standard deviation. n.s., not significant.

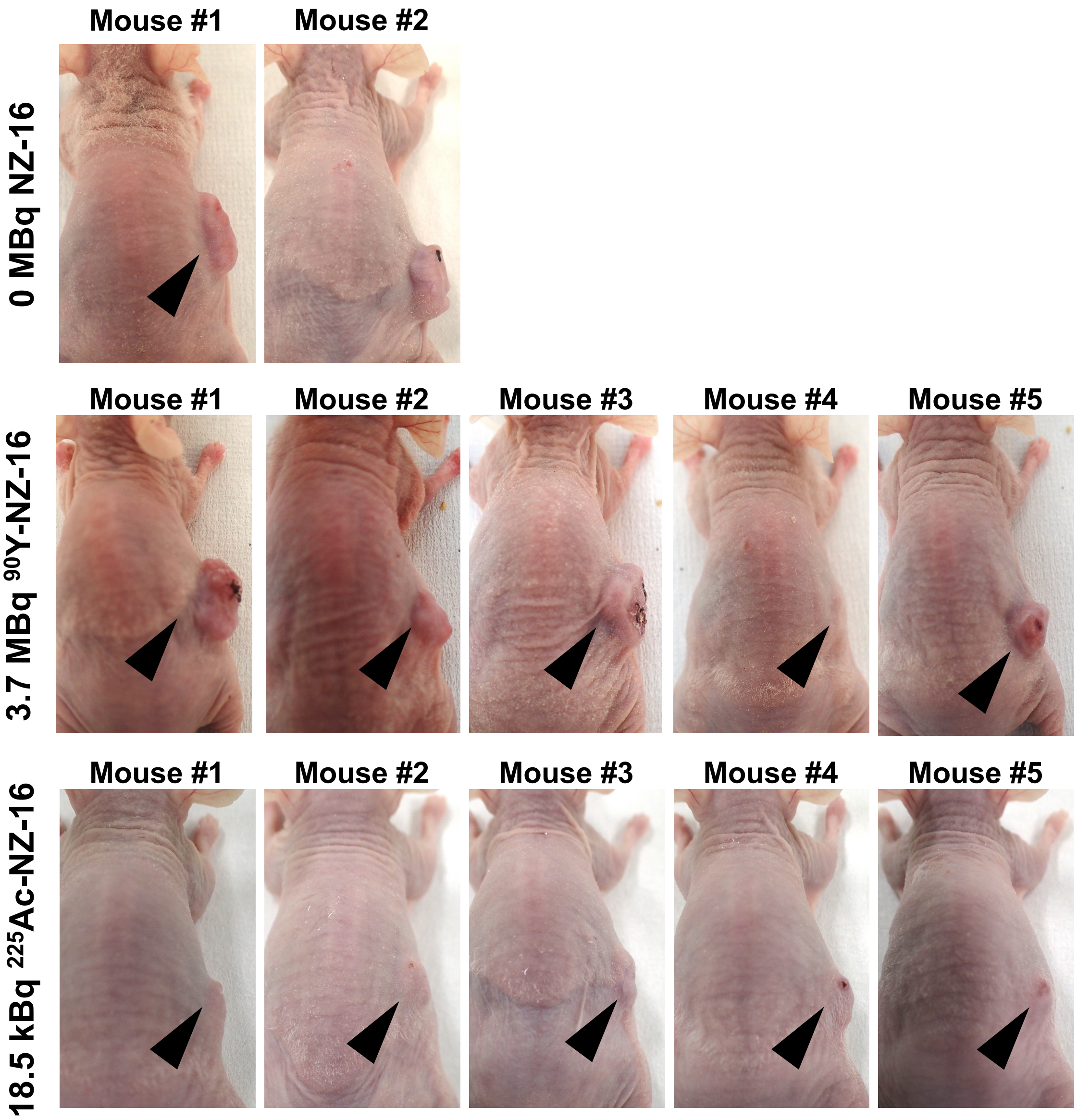

Supplementary Figure S3. Photos of mice treated with 0 MBq, 3.7 MBq of 90Y-labeled NZ-16 and 225Ac-labeled NZ-16 at Day 56. Arrowheads indicate H226 tumors.

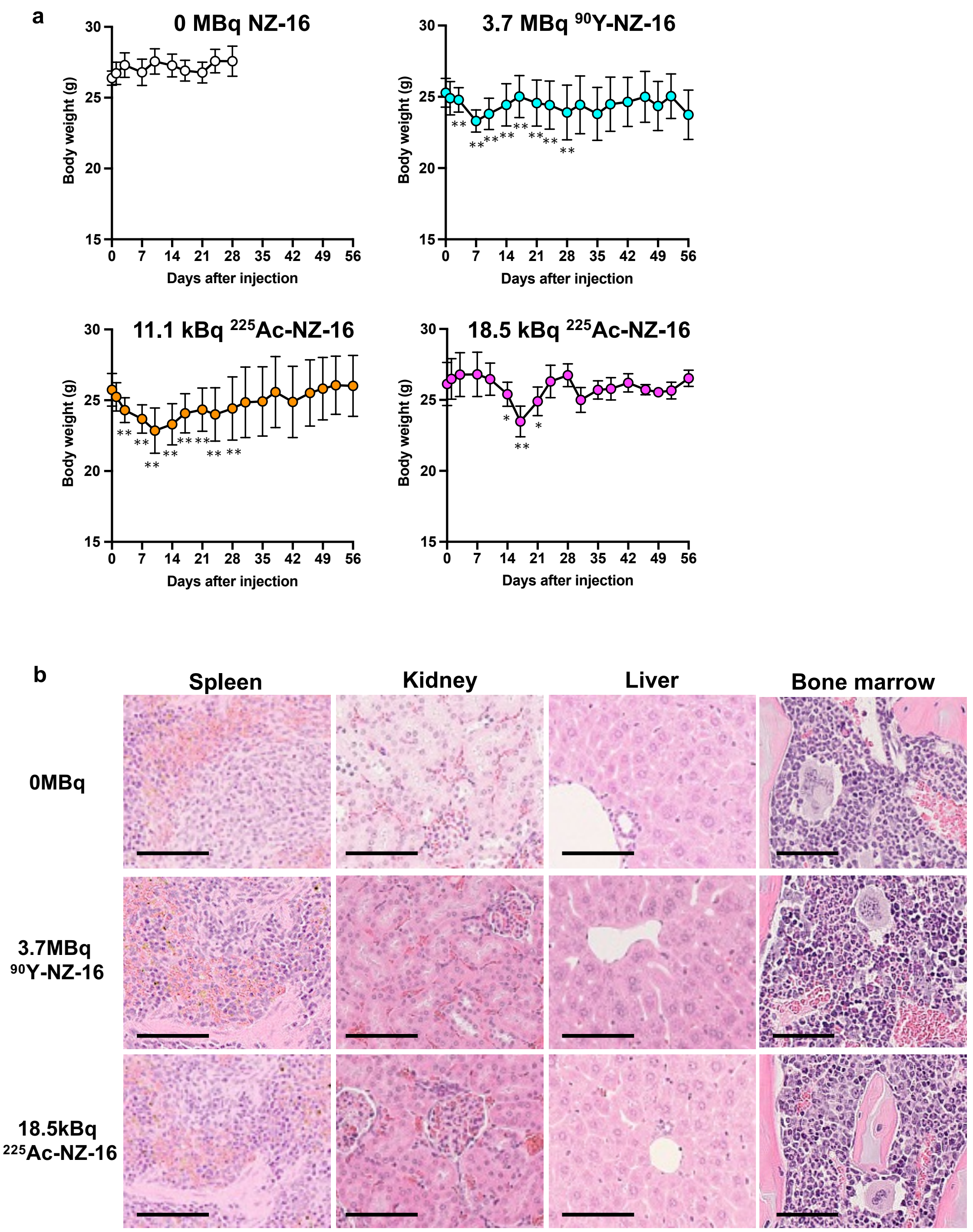

Supplementary Figure S4. Side effects after treatments. (a) Body weight changes after injection with ^{90}Y- and ^{225}Ac-labeled NZ-16. Data indicate mean and standard deviation (n = 5). **P* < 0.05, ***P* < 0.01 vs. 0 MBq NZ-16. (b) H&E-stained sections of spleen, kidney, liver, and bone marrow. Scale bar, 50um.

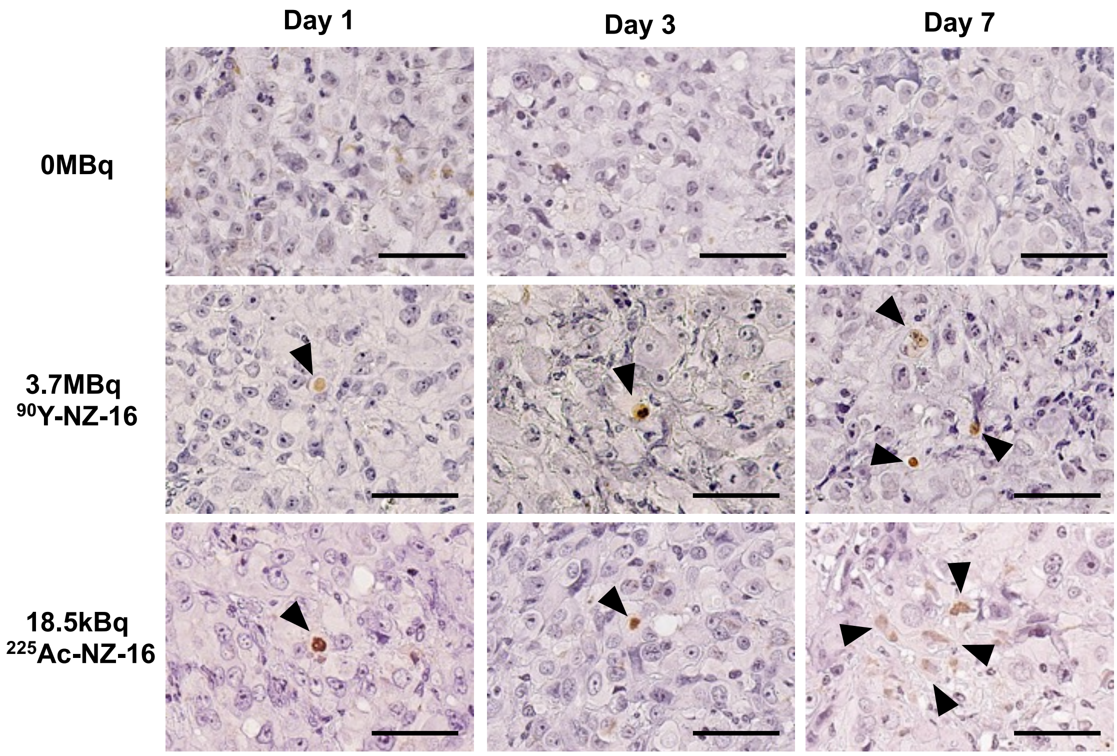

Supplementary Figure S5. TUNEL-stained H226 tumors at days 1, 3, and 7 after injection with 0 MBq (intact NZ-16 only), 3.7 MBq of ^{90}Y-labeled NZ-16, and 18.5 MBq of ^{225}Ac-labeled NZ-16. Arrowheads indicate TUNEL positive cells. Bar, 50 µm
